# Osteoblast-like Cell Proliferation, ALP Activity and Photocatalytic Activity on Sintered Anatase and Rutile Titanium Dioxide

**DOI:** 10.3390/ma14164414

**Published:** 2021-08-06

**Authors:** Yukiko Yokoi

**Affiliations:** Department of Dental Materials, School of Dentistry, Matsumoto Dental University, 1780 Hiro-Oka Gobara, Shiojiri, Nagano 399-0781, Japan; yukiko.yokoi@mdu.ac.jp

**Keywords:** titanium dioxide (TiO_2_), anatase, sintered, cell proliferation, alkaline phosphatase (ALP)

## Abstract

This study aimed to create a biomaterial from titanium dioxide (TiO_2_), which has been known to have photocatalytic and bone formation promoting effects. I expected that anatase titanium dioxide-based implants could promote bone augmentation and induce bone formation. Powdery anatase TiO_2_ was compression molded and sintered at 700, 800, 900, and 1000 °C to prepare sintered compact samples. X-ray diffraction and scanning electron microscopy were used to observe the surface of these samples. Furthermore, mouse osteoblast-like cells (MC3T3-E1 cell line) were seeded on the samples sintered at different temperatures, and cell proliferation was observed to evaluate the cell proliferation of the samples. The sample sintered at 700 °C was composed of anatase TiO_2_. The samples sintered at 800 °C and 900 °C were confirmed to consist of a mixture of anatase and rutile TiO_2_ crystalline phases. Moreover, the sample sintered at 700 and 800 °C, which contained anatase TiO_2_, showed remarkable photocatalytic activity. Those samples sintered at 1000 °C were transformed to the rutile TiO_2_. The cell proliferation after 7–14-days culturing revealed that cells cultured on the 700 °C sample decreased in number immediately after initiation of culturing. The cells cultured on TiO_2_ sintered at 900 °C markedly proliferated over time with an increase in the alkaline phosphatase activity, showing good MC3T3-E1 cell compatibility of the samples. The sample sintered at 1000 °C, which is rutile TiO_2_, showed the highest increase.

## 1. Introduction

Pure metallic titanium and titanium alloys are applied as dental biomaterials, for instance, as implants, and it is considered that their satisfactory biocompatibility depends on the passivated coating film of titanium dioxide (TiO_2_) formed on the surface of the implant. However, TiO_2_ is a ceramic and a chemically stable material that is not damaged by an acid or alkali at ordinary temperature. It has long been used as a raw material for white paints, pigments, material for porcelain, abrasives, pharmaceuticals, and cosmetics products [1,2]. It is also the starting material for smelting metallic titanium [3]. Titanium dioxide exists in three types of crystal structures, namely, tetragonal rutile and anatase, and orthorhombic brookite. The rutile is stable over the whole temperature range from normal temperature to the melting point. The anatase irreversibly transforms into the rutile at 915 °C. The brookite type has almost no practical application because of the difficulty to form pure crystals.

Past research revealed that the surface of a TiO_2_ thin film formed by depositing ceramic TiO_2_ on metallic titanium by direct current magnetron sputtering method exhibits good quality, homogeneity, roughness and biocompatibility [4]. There is, however, little research on the biocompatibility of the ceramic TiO_2_.

On the rutile TiO_2_, the growth of osteoblast-like MC3T3-E1 cells and the alkaline phosphatase (ALP) activity has been investigated in the preceding article [5]. Moreover, it was clarified that the rutile TiO_2_ sintered at 1300 °C showed good biocompatibility and increase in ALP activity. On the other hand, anatase TiO_2_ has a photocatalytic effect and high antibacterial activity [6], but it has not been investigated from the standpoint of a bone grafting or implant material. As the component ratio of anatase and rutile phases in TiO_2_ changes depending on the sintering temperature, it may be possible to create TiO_2_ with the properties of both phases.

In the present study, to develop biomaterials having both photocatalytic function and biocompatibility, anatase TiO_2_ powder was sintered at various temperatures, and its cell proliferation, ALP activity, and photocatalytic activity were measured using osteoblast-like MC3T3-E1 cells. Moreover, the compatibility of the sintered TiO_2_ as an osteoinductive biomaterial was examined. This is a pilot study for producing multifunctional biomaterials based on TiO_2_.

## 2. Materials and Methods

### 2.1. Sample Preparation

Anatase TiO_2_ powder (ST-01, Ishihara Sangyo Kaisiha, LTD., Osaka, Japan) with an average size of 7 nm of primary particles was used. To prepare the molded product, 0.5 g of TiO_2_ and 0.3 mL of distilled water were weighed and mixed, filled in a metal mold with 15 mm inner diameter, and a load of 15 kN was applied for 1 min to prepare a sample of 3 mm in thickness. The molded sample was heated in an electric furnace (LP-907, Koyo Thermo System Co., Ltd., Nara, Japan) from room temperature to 100 °C for 30 min, and after drying, sintered by heating to a predetermined sintering temperature for 120 min. The samples were sintered in the atmosphere at a temperature of 700 to 1000 °C, followed by anchoring at a predetermined temperature, cooling in the furnace and used for the experiments. Four samples were prepared for sintering temperatures 700, 800, 900, and 1000 °C. The samples were ultrasonically washed with ethanol followed by distilled water and then sterilized in autoclave at 120 °C for 15 min.

### 2.2. X-ray Diffraction of TiO_2_ Sintered Body

X-ray diffraction was performed to clarify the composition of the sintered TiO_2_. The crystal phases of the starting material and the TiO_2_ sintered bodies sintered at 700, 800, 900, and 1000 °C were identified using an X-ray diffractometer (Rad-rC, Rigaku Corporaton, Tokyo, Japan). The measurements were performed under conditions of tube voltage of 40 kV, tube current of 60 mA, bulb CuKα and a Ni filter with a diffraction angle 20 to 60°.

### 2.3. Surface Observation of TiO_2_ Sintered Body

The surface characteristics of TiO_2_ after sintering at each sintering temperature were observed with a scanning electron microscope (SEM) (JSM-6000, JEOL Ltd., Tokyo, Japan). The acceleration voltage was set at 20 kV.

### 2.4. Cell Growth on TiO_2_ Sintered Body

Using mouse osteoblast-like MC3T3-E1 cells (KAC Co., Ltd., Kyoto, Japan), the culture solution was α-Minimum Essential Medium (GIBCO, Thermo Fisher Scientific Inc., Waltham, MA, USA) with the addition of 10% fetal bovine serum (EQUITECH-BIO, Kerrville, TX, USA), 100 U/mL Penicillin (GIBCO, Thermo Fisher Scientific Inc., Waltham, MA, USA), and 100 μg/mL Streptomycin. A TiO_2_ sample was placed in a 24-well microplate (Falcon), the cell suspension was adjusted to 2.0 × 10^4^ cells/mL and 600 μL was seeded in each well. Culturing was performed in 5% CO_2_ at 37 °C for 7 and 14 days. The number of cells was measured by transferring each sample to a new 24-well microplate containing 600 μL of culture solution. Subsequently, 120 μL of CellTiter 96^®^ AQueous One Solution Reagent (Promega, Madison, WI, USA) was added to each well, and after culturing for 2 h, the absorbance (wavelength 492 nm) was measured using a microplate reader (MPR—A4i, Tosoh, Tokyo, Japan). The ALP activity was measured via enzymatic reaction of the samples after culturing on the 7 and 14 days with a TRACP&ALP Assay Kit (Takara Bio Inc., Tokyo, Japan) at 37 °C for 30 min and measuring the absorbance (wavelength 405 nm) using a microplate reader. As the behavior of cell on the anatase sample was observed compared to the sample sintered at 1000 °C, which was transformed to the rutile. Celldesk LF1 (Sumitomo Bakelite Co., Ltd., Tokyo, Japan) polystyrene film dish was used as the control material. Five samples were prepared for each culturing day.

### 2.5. Cell Morphology Observation

The cell morphology was observed on each sample 7 days after the culturing. The samples were immobilized in 99.8% methanol (Kanto Chemical Co., INC., Tokyo, Japan for 10 min, after airdried, immersed for 30 min in Giemsa’s stain (Merck, Darmstadt, Germany). Moreover, it was diluted to 20% with distilled water, after washed with running water, airdried. An inverted metallurgical microscope (PME3, Olympus Corporation, Tokyo, Japan) was used to observe the cells on the sample.

### 2.6. Photocatalytic Activity

Photocatalytic oxidation is considered to be one of the most effective ways of degrading methylene blue. The catalytic activity of TiO_2_ was evaluated based on the dye resolution, in which the sample was immersed in 1 mL of aqueous methylene blue solution and placed at 25 cm distance from visible light. After 24 h, optical absorbance of the solution was measured at a wavelength of 595 nm. Absorbance of the untreated methylene blue solution (control) was set as 100%, and the dye resolution was defined by subtracting the absorbance value (%) from 100.

### 2.7. Statistical Processing

For each measured value, statistical analysis was performed by one-way analysis of variance, followed by multiple comparison tests using Tukey’s method.

## 3. Results

### 3.1. Identification of Crystal Phase

**Figure 1:**
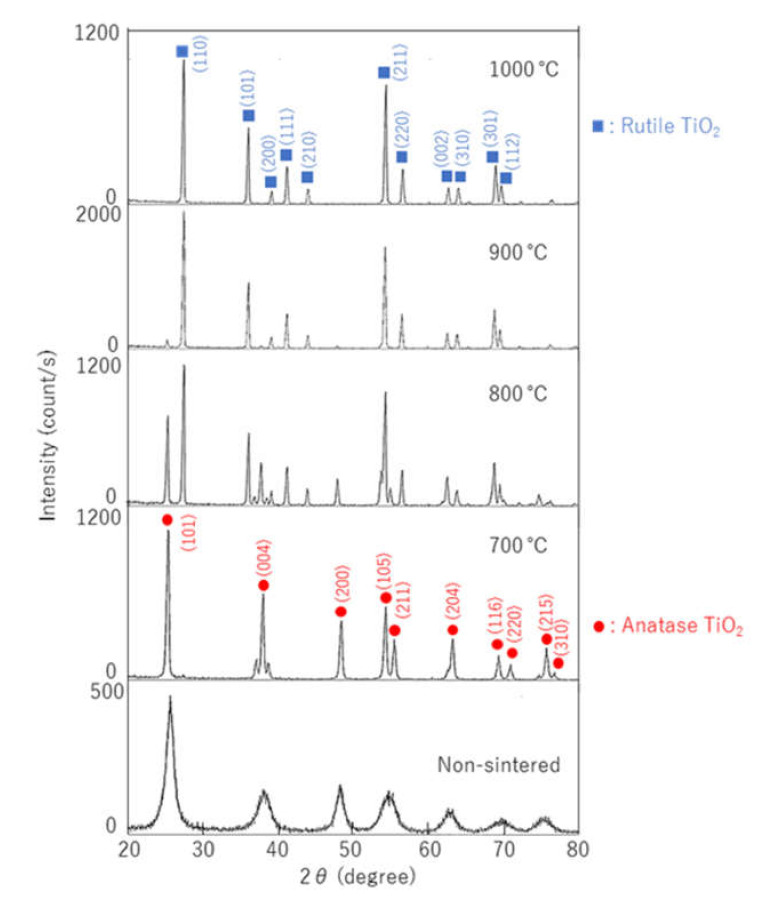
X-ray diffraction patterns for TiO_2_ sintered at different temperatures (700, 800, 900, and 1000 °C). Diffraction patterns for anatase and rutile TiO_2_ are based on PDF of ICDD.

The X-ray diffraction patterns of the samples are illustrated in Figure 1, in which diffraction patterns with Miller indices for anatase and rutile TiO_2_ are written based on PDF of ICDD. Only anatase TiO_2_ could be identified in the non-sintered sample and that sintered at 700 °C. Anatase and rutile phases were observed in the sample sintered at 800 °C. In the sample sintered at 900 °C, the rutile and slight anatase phases were observed. Only the rutile phase was observed in the sample sintered at 1000 °C. As the sintering temperature increased, the crystal phase of TiO_2_ changed gradually from anatase to rutile. This phase change was completed at 1000 °C.

### 3.2. Surface Observation

**Figure 2:**
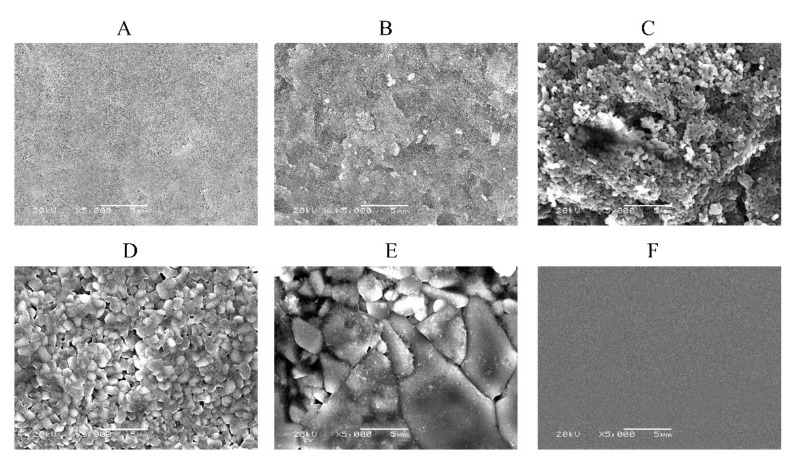
SEM images: (**A**) Unsintered TiO_2_; (**B**) TiO_2_ sintered at 700 °C; (**C**) TiO_2_ sintered at 800 °C; (**D**) TiO_2_ sintered at 900 °C; (**E**) TiO_2_ sintered at 1000 °C; (**F**) Control (Celldesk LF1, Polystyrene film dish). Scale bar = 5 μm for all the images.

Figure 2 shows the surface texture of the samples and the control taken with the SEM. The shape and size of the crystal particles in each sample differed depending on the sintering temperature. In the non-sintered sample, TiO_2_ particles whose initial sizes were 7 nm were pressed and relatively smooth. In the samples sintered at 700 and 800 °C, the clearly sintered structure could not be observed. When rising the sintering temperature above 900 °C, the particles began to contact with each other. After that, the contact surface increased and grain growth advanced with the formation of many pores between particles. In the sample at sintered at 1000 °C, fusion of crystals of about 5 μm is observed, which resulted in irregular grain growth. The higher the sintering temperature, the more remarkable was the grain growth.

### 3.3. Cell Growth and Alkaline Phosphatase Activity

**Figure 3:**
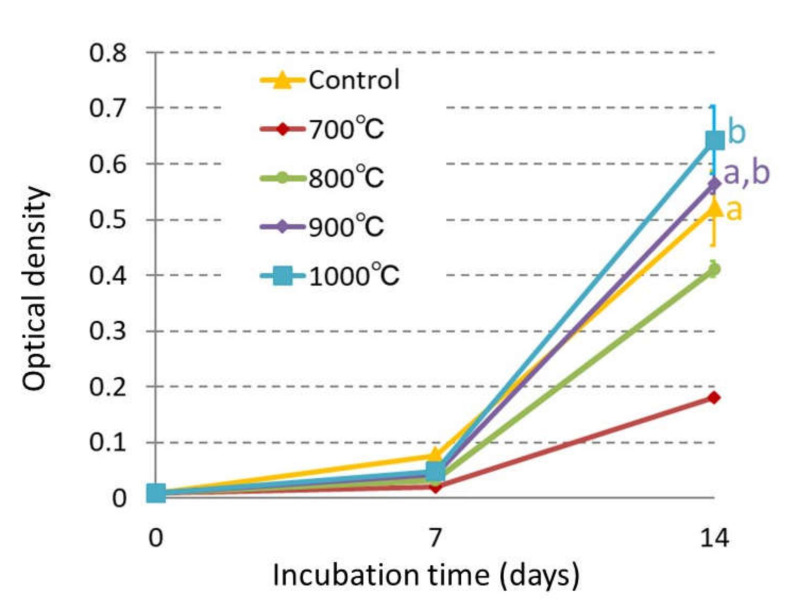
Cell proliferation of MC3T3-E1 cells on 700, 800, 900, and 1000 °C TiO_2_ samples and Celldesk LF1 (Polystyrene film dish). Optical density is equivalent to the number of cells. A significant difference in cell proliferation was identified every group on day 7 of culture (*p* < 0.05). On day 14, with the same letter indicating that there is no significant difference between the groups (*p* < 0.05).

**Figure 4:**
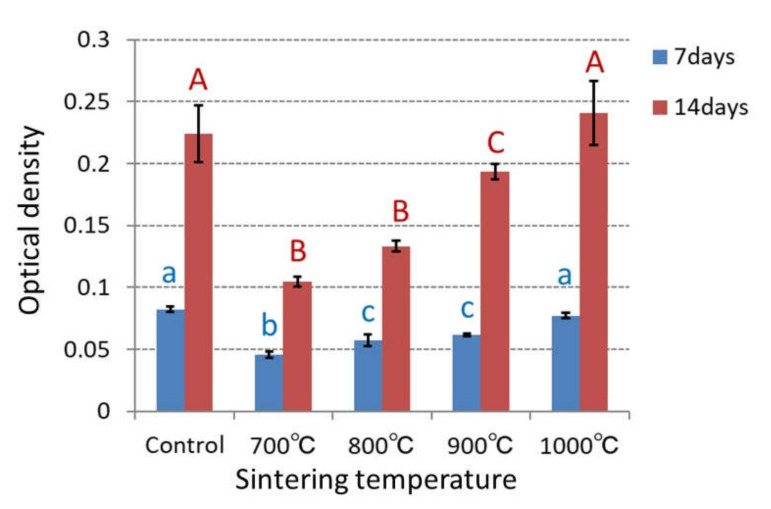
ALP activity of cells on 700, 800, 900, and 1000 °C TiO_2_ samples, which is indicated by optical density measured with microplate reader. With the same letter indicating that there is no significant difference between the groups (*p* < 0.05).

The ability of anatase TiO_2_ as an osteoinductive biomaterial was investigated by the proliferation and ALP activity of MC3T3-E1 cells, which were cultured on the sample for 7, 14 days. The cell growth and ALP activity were indicated by the optical density measured with the microplate reader (Figure 3 and Figure 4). 

After 7 days of culturing, the cell growth in the samples sintered at 700 to 900 °C decreased compared to the control. After 14 days, the cell growth increased with the sintering temperature. In the samples sintered at 700 and 800 °C, the cell growth after 14 days was significantly lower than those of the other samples (*p* < 0.05). However, in the samples sintered at 900 and 1000 °C, the cell growth increased to the same level as the control. 

After 7 and 14 days, the ALP activity increased with the sintering temperature. In the sample sintered at 1000 °C, after 7 days, the ALP activity was slightly lower than that in the control. However, after 14 days, it became higher than that in the control. Increasing ratio of the ALP activity with the sintering temperature after 14 days was larger than that after 7 days.

### 3.4. Cell Morphology Observation

**Figure 5:**
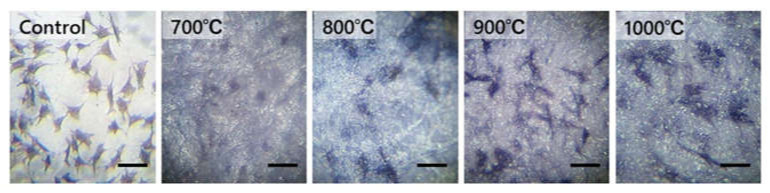
Images of cells on each sample TiO_2_ sintered at 700, 800, 900, and 1000 °C and control (Celldesk LF1; Poly styrene film dish). Scale bar = 40 μm for all the images.

Figure 5 shows the morphology of MC3T3-E1 cells for 7 days of culturing. On the sample sintered at 700 °C, the number of cells was smaller than that on the other samples, which showed scattered images. On some samples, signs of atrophy were observed in their morphology. However, spindle-shaped, triangular, and spherical cells were observed on the samples sintered above 800 °C. The cells were either polygonal or circular, and protrusions were observed to radiate around the cells and attach to the sample surface, giving substantially the same shape as the control.

### 3.5. Photodegradation of Methylene Blue

The photocatalytic activity of each TiO_2_ specimen was evaluated by the dye resolution (degradation) of methylene blue aqueous solution (Table 1). When rising the sintering temperature, the dye resolution decreased. In the sample sintered at 700 and 800 °C, the dye resolutions were 73.3 and 72.4%, respectively, which showed remarkable photocatalytic activity. In the sample sintered at 900 °C, the dye resolution was 60.9%, which had sufficient photocatalytic activity compared to that at 1000 °C.

## 4. Discussion

### 4.1. Application of Titanium Dioxide to Biomaterials

Titanium dioxide is extensively used in medical applications for its excellent biocompatibility and high mechanical strength [7]. Crystalline titanium dioxide exists in three phases: anatase, rutile, and brookite. Both anatase and rutile can form bioactive hydroxyapatite layers in vitro and have outstanding biocompatibility [7]. Owing to their compatibility, the rutile and anatase TiO_2_ surfaces can function as substrates for the growth of different cell types [8,9,10,11,12]. Furthermore, it has been reported that long term culturing on rutile and anatase TiO_2_ films induces the growth of hepatocytes and sustains metabolic activity [12,13]. Studies on CHO-K1 cells cultured on anatase and anatase/rutile TiO_2_ thin films that evaluated the cytotoxic effect of TiO_2_ thin films demonstrated that CHO-K1 cells displayed growth that was as good as that of the control group. This indicates that anatase and anatase/rutile TiO_2_ thin films have no adverse effect on cell viability and growth. Moreover, it has been suggested that a TiO_2_ thin film sintered at 800 °C offers the optimum conditions for the ratio of anatase to rutile regarding the survival and growth of CHO-K1 cells [4].

Similarly, in this study, to create an anatase-only and anatase-rutile mixed samples, particles of anatase TiO_2_ were compressed and sintered at 700 to 900 °C. Cells were cultured on the samples and their growth and ALP activities were measured. Their results showed that the mixed samples of anatase and rutile clearly enhanced the growth of cells more than anatase alone. The cell growth and ALP activities were maximized in the rutile-only sample sintered at 1000 °C.

### 4.2. Mold Ability, Sintering, and Surface Characteristics of Titanium Dioxide

Sintering is a molding method for making a powdered solid into a desired shape by heating and compression, in which a durable material is produced through the sticking together of the particle. Rutile and the anatase TiO_2_ are solid phase sintered substances, and no liquid phase exists at the sintering temperature. Densification occurs because of a morphological change due to the coalescence of particles and it advances through the diffusion [14,15]. In a general property of ceramics, when uprising the sintering temperature, the grain size increase and reduces the pores [16,17]. The increase in grain size is caused by expediting the movement of grain boundary, while its detailed mechanism could not be examined from the present results alone. Because transformation to the rutile occurs at 800 to 1000 °C, the sintering temperature was set to 700 to 1000 °C in consideration of the photocatalytic activity and the mechanical properties. As the primary particles of the anatase TiO_2_ were as fine as 7 nm, the sintering occurred even at low temperatures due to high sintering velocity and high density of the samples.

Increase in the hardness with higher sintering temperature is believed to be due to the sintering process. The higher the sintering temperature was set, the greater the amount of grain enlargement exhibited.

### 4.3. Change in ALP Activity and Photocatalytic Activity with Sintering Temperature

In all TiO_2_ samples, ALP activity increased during 7 to 14 days of incubation. After 14 days of incubation, all samples showed more than double ALP activity compared to 7 days. In particular, at sintering temperatures 900 °C and 1000 °C, the increase in ALP activity showed 3.1 times, which was larger than 2.7 times in the control. This was because the rutile TiO_2_ promoted ALP activity.

When sintering at 700 and 800 °C, the dye resolution was almost the same as in the non-sintered sample. This is due to the presence of anatase TiO_2_, which was demonstrated by the X-ray diffraction analysis. In an initial state, OH-groups are bound to the surface of anatase TiO_2_ and strong catalytic action was shown. Preliminary experiments were conducted without light irradiation, but there was no significant difference between with and without light irradiation, and the same catalytic activity was observed.

When sintering at 900 °C, the X-ray diffraction analysis revealed the presence of both the rutile and the anatase TiO_2_, and the dye resolution was 60.9% after 24 h. The sample sintered at 1000 °C, which was only rutile TiO_2_, the dye resolution (29.1%) diminished compared to the samples sintered at 700 to 900 °C.

Anatase TiO_2_ has higher photocatalytic activity than rutile TiO_2_ [6]. Therefore, the catalytic activity was high at the sintering temperatures 700 and 800 °C due to the anatase TiO_2_, and very low at 1000 °C due to the rutile TiO_2_. (Table 1)

In this experiment, the photocatalytic activity of each sample was measured 24 h, but it is also necessary to examine the short-term changes, for instance, at every 30 min. It is difficult to measure the reaction rate, and the present method for measuring the rate of dye discoloration may cause change in color even in the absence of oxidation reaction [17]. To evaluate the catalytic reaction in detail, future studies will be necessary.

### 4.4. Evaluation of Titanium Dioxide Sintered Body as a Biomaterial

Ceramic state TiO_2_ is an extremely stable substance and both its anatase and rutile are dissoluble in hydrofluoric acid, sulfuric acid and hydrochloric acid, but not in other substances.

The aim was to develop biomaterials having both photocatalytic function and biocompatibility. When sintering at 900 °C, a combined TiO_2_ of anatase and rutile phases was created, and it had good proliferation and ALP activity, and also high photocatalytic activity. Although further verifications must be necessary, this TiO_2_ may be expected as a multifunctional biomaterial with biocompatibility and antibacterial property.

There are reports on the photocatalytic effect of the anatase TiO_2_ [18] and its efficiency in bactericidal action [19], on cell dynamics of a material coated with TiO_2_ [20,21,22,23,24,25,26] and on cytotoxicity caused by super-particle size anatase TiO_2_ [27,28]. However, there are few reports on sintered anatase TiO_2_ [29,30,31].

In this experiment, mouse osteoblast-like MC3T3-E1 cells were used to evaluate the biocompatibility of TiO_2_. This method has been used for evaluating dental biomaterials, in which the cell growth offers a highly reproducible and rapid evaluation [15,16,17]. The ALP activity is an index of osteoblast-like cell differentiation that enables evaluation whether or not the osteoblast cells are active. There are few reports to evaluate the biocompatibility of TiO_2_ as a ceramic as in this experiment [25].

In the sample sintered at 700 °C, strong catalytic activity was shown, while the increase in proliferation and ALP activity was modest. In the sample sintered at 800 °C, moderate proliferation and ALP activity was observed with the lapse of time, but lower than those of the control. However, good proliferation was observed in the sample sintered at 900 °C. This phenomenon can be probably attributed to the transformation of the anatase into rutile TiO_2_. Meanwhile, when sintering at 700 °C, the anatase only sample was created, and thereby the proliferation was suppressed. This sample showed strong photocatalytic activity, because the primary particles of anatase TiO_2_ were as small as 7 nm and had a large surface area, on which OH-groups were bonded already in the initial state. These results indicate that strong radical generation and oxidizing power decomposed the organic components of cells or in the culture solution, and thereby the cell growth was suppressed.

In the sample sintered at 900 °C, the X-ray diffraction analysis identified both the rutile and the anatase TiO_2_, although their composition ratio was not determined. This sample showed good proliferation and sufficient photocatalytic activity. This was due to the rutile phase transformed from the anatase TiO_2_ during sintering, in which the catalytic activity was reduced but the radicals and oxidizing power remained below the level that does not affect the cell affinity. Only the rutile TiO_2_ sintered at 1000 °C had the best cell compatibility. In the case of full transformation into the rutile TiO_2_, the authors have already reported that cell compatibility was shown and the catalytic activity was reduced [5]. This is because a single crystal of the rutile TiO_2_ has a simple structure, exhibits low catalytic activity and is chemically equivalent and stable. Anatase TiO_2_ is higher cytotoxic compared to rutile TiO_2_ [6]. At sintering temperature 700 °C, because the crystal phase was anatase, the proliferation was inhibited, and the number of cells increased only slightly in Figure 3. On the other hand, the anatase TiO_2_ was transformed completely to the rutile TiO_2_ at sintering temperature 1000 °C, thereby the cell proliferation was maximized [32]. 

The above results clearly demonstrated that the anatase TiO_2_ can impart a gradient function to the rutile TiO_2_, that is, the biocompatibility and catalytic activity of TiO_2_ can be modified by changing the sintering temperature. This finding was derived from the experiments using MC3T3-E1 cells, and currently, I am investigating its relationship with mouse-derived osteoblasts.

By performing XRD interpretation with the TEM and HR-TEM, the microstructure of the combined TiO_2_ of anatase and rutile phases may be identified, and it may be possible to examine the detail mechanisms of proliferation and ALP activity. This remains an issue to be pursued in the future.

## 5. Conclusions

It was found that cell proliferation, ALP activity, and photocatalytic activity changed depending on the crystal types anatase and rutile TiO_2_.

When sintering at 900 °C, composite of anatase and rutile TiO_2_ was created, which showed good proliferation and high ALP activity, and sufficient photocatalytic activity. This TiO_2_ was expected as a multifunctional biomaterial. But further studies are needed for validating their functions.

## Figures and Tables

**Table 1:** Catalytic activity (dye resolution) of TiO_2_ at various sintering temperatures.

Sintered Temperature	700 °C	800 °C	900 °C	1000 °C
Dye resolution	73.3%	72.4%	60.9%	29.1%
Degradation color of methylene blue solution	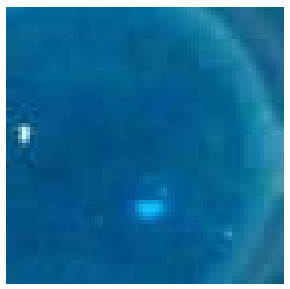	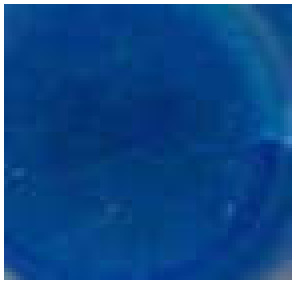	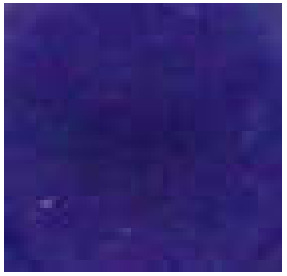	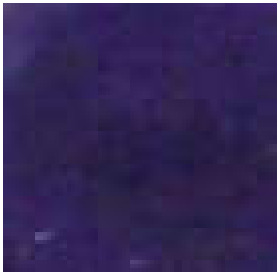

## Data Availability

Not applicable.
